# Rational Construction of a Responsive Azo-Functionalized Porous Organic Framework for CO_2_ Sorption

**DOI:** 10.3390/molecules26164993

**Published:** 2021-08-18

**Authors:** Rongrong Yuan, Hao Sun, Hongming He

**Affiliations:** 1 Department of Materials Science and Engineering, Jilin Jianzhu University, Changchun 130118, China; yuanrongrong@jlju.edu.cn (R.Y.); sh2456755@163.com (H.S.); 2 Tianjin Key Laboratory of Structure and Performance for Functional Molecules, College of Chemistry, Tianjin Normal University, Tianjin 300387, China

**Keywords:** porous organic framework, azo, stimuli-responsive, CO_2_ sorption

## Abstract

An azo-functionalized porous organic framework (denoted as JJU-1) was synthesized via FeCl_3_-promoted oxidative coupling polymerization. By virtue of a porous skeleton and a light/heat responsive azo functional group, this task-specific JJU-1 displays a reversible stimuli-responsive adsorption property triggered by UV irradiation and heat treatment. The initial Brunauer–Emmet–Teller (BET) surface area of this porous material is 467 m^2^ g^–1^. The CO_2_ sorption isotherms exhibit a slight decrease after UV irradiation because of the *trans* to *cis* conversion of the azo functional skeleton. It is worth mentioning that the responsive CO_2_ adsorption performance can be recycled for three cycles via alternating external stimuli, confirming the excellently reversible switchability of *trans*-to-*cis* isomerization and controllable CO_2_ adsorption.

## 1. Introduction

As a common greenhouse gas, the increased concentration of carbon dioxide (CO_2_) has caused global ecological and environmental problems. Effective capture and utilization of CO_2_ is very important for environmental protection and economic value. To settle this thorny problem, one of the most effective approaches is the carbon capture and storage (CCS) technology [1,2]. Recently, many scientists have developed and exploited various materials for high-efficient adsorption of CO_2_ [3,4,5,6]. In the last few decades, porous organic frameworks (POFs) have been attracting a great deal of attention, because they have many advantages including a high surface area, excellent physicochemical stability, convenient designability, and fascinating structure [7,8,9,10,11,12]. POFs have been synthesized in large quantities and widely investigated in the CO_2_ sorption field [13,14,15,16]. Up to now, various synthetic reactions have been explored in the preparation of POFs, including the Yamamoto reaction [17], the Sonogashira–Hagihara cross-coupling reaction [18], the Suzuki coupling reaction [19], oxidative coupling polymerization [20], and the Friedel–Crafts alkylation reaction [21]. Nevertheless, most polymerization reactions commonly require rigorous reaction conditions in the preparation of POFs, including high temperatures, expensive noble metal catalysts, and inert gas shielding. Recently, the FeCl_3_-promoted oxidative coupling polymerization has been considered as a potential approach to construct POFs [22,23,24,25,26]. This method possesses outstanding advantages due to the low-cost catalyst, moderate reaction temperature, and high yield. Among all organic monomers, carbazole-based building blocks are easily to construct three-dimensional (3D) porous structures. Meanwhile, carbazole itself has good thermal stability and acid/alkali resistance. Its unique rigid ring and conjugated electron-rich system are not only beneficial to the formation of porous materials, but also can strengthen the interaction between carbazole and the adsorbed substance. The electron-rich carbazole groups can be coupled chemically under an oxidant such as FeCl_3_ [27,28,29].

Recently, photoresponsive CO_2_ adsorbents have been attracting lots of attention, because the CO_2_ adsorption capacity can be adjusted conveniently by light irradiation. As we know, functional materials that respond to different external stimuli, such as redox potential, temperature, pH value, and light, have been developed and used extensively in various applications [30,31,32,33,34,35]. The development of sensitive smart materials has become one of the important research areas, because the controllability, rapid responsiveness, and high precision are beneficial in many vital applications. Light is considered as the most attractive among all stimulation forms, because light is a handy and usually non-invasive signal. Photochromic molecules like azobenzene have been attracting particular attention for photoresponsive functional materials [36]. Upon UV irradiation, azobenzene can isomerize from the nonpolar and planar *trans*-form to the nonplanar *cis*-form with a dipole moment, which can significantly affect the sorption and release of guest species [37]. Additionally, the *cis* azobenzene can go back to its thermodynamically stable *trans*-form via the irradiation with visible light or thermal relaxation. Recently, the azobenzene photoresponsive porous materials have been designed and explored for regulating CO_2_ adsorption performance [38,39,40,41,42,43,44,45]. It is still very important to design and prepare such functional materials with CO_2_ storage/release sensitivity and good CO_2_ separation performance.

**Scheme 1:**
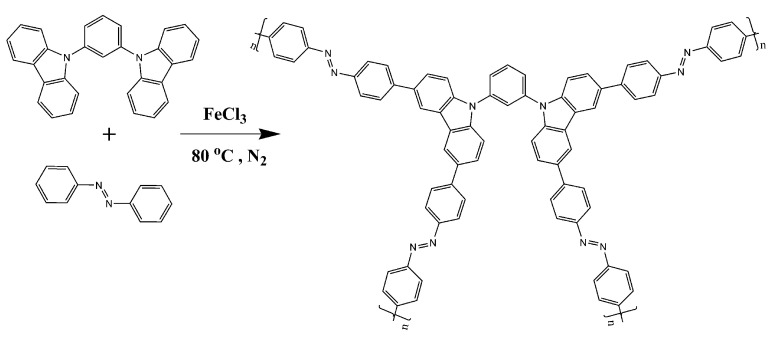
The synthetic process of JJU-1 via FeCl_3_-promoted oxidative coupling polymerization.

Taking the above into account, a carbazole-based organic building block of 1,3-*bis*(N-carbazolyl)benzene (BCB) was selected to react with azobenzene (AB) to construct a novel POF material (denoted as JJU-1) by FeCl_3_-promoted oxidative coupling polymerization (Scheme 1). The as-synthesized JJU-1 has high thermal stability, moderate surface area, and hierarchical pore structure. Furthermore, the CO_2_ adsorption isotherms of JJU-1 under UV irradiation and thermal regeneration can be well-repeated for three cycles. The CO_2_ uptake corresponds to a 11.5% decrease after UV irradiation, and almost recovers to the initial value after thermal regeneration, indicating the reversibility of the photoresponsive behavior in this prepared porous material. Therefore, the azo-functionalized POF with controllable CO_2_ adsorption could bring potential applications in CCS.

## 2. Results

### 2.1. Structural Description

**Figure 1:**
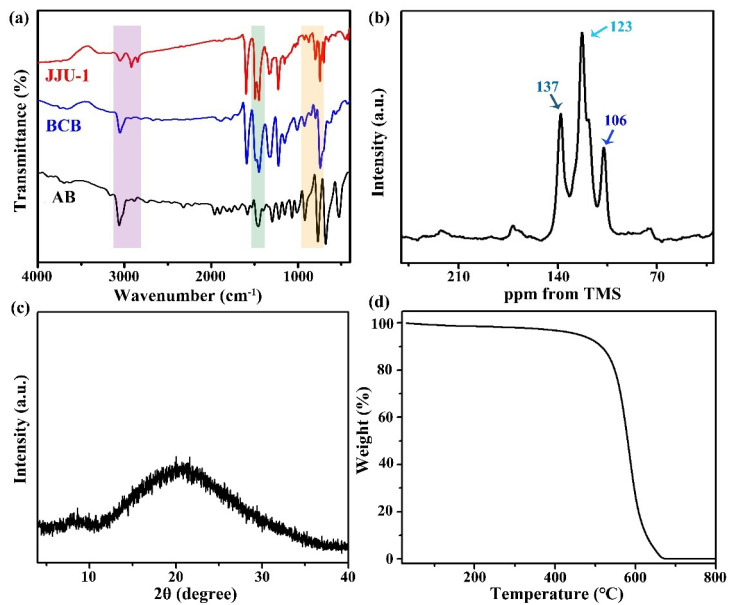
(**a**) FT-IR spectra of AB (black), BCB (blue) and JJU-1 (red); (**b**) the ^13^C solid-state NMR spectrum; (**c**) PXRD and (**d**) TGA curve of JJU-1.

Firstly, the *trans*/*cis* isomerization of azobenzene as a research model is confirmed by UV-Vis measurements. A typical strong π→π* absorption band at 325 nm and a weak π→π* absorption band at 435 nm in dichloromethane can be found in the UV-Vis spectra, which are assigned to the *trans* and *cis* isomerization of the azo functional group. After exposure to UV light, the intensity of the 325 nm band decreases along with the slightly increasing intensity of the 435 nm band because of the *trans*-to-*cis* isomerization of azobenzene (Figure S1). Furthermore, the prepared sample and the UV/heat irradiated samples are fully studied by many technologies. The skeleton structure of JJU-1 is initially investigated by Fourier transform infrared (FT-IR) spectroscopy (Figure 1a). Through comparison of the FT-IR spectra of initial monomers and final products, the characteristic absorption peaks are summed up as follows: (a) an obvious peak at ~3000 cm^–1^ is mainly ascribed to the C–H stretching vibration of the hydrogen atom in phenyl ring; (b) the peaks (680~560 cm^–1^) belong to the C–H deformation vibration of four adjacent hydrogen atoms in the 1,2-disubstituted phenyl ring of carbazole group, which are significantly weakened due to the reduction of adjacent ring hydrogen atoms from four to three; (c) some peaks in the range of 800~690 cm^–1^ are the C–H deformation vibration of ring hydrogen atoms of the 1,2-disubstituted phenyl ring of the carbazole group; (d) the characteristic stretching band of –N=N– at 1445 cm^–1^ presents in JJU-1 [46]. Furthermore, the ^13^C solid-state NMR was measured to determine the skeleton structure of JJU-1. As seen in Figure 1b, three prominent peaks are present in the range of 100~150 ppm, belonging to aromatic carbon atoms of the phenyl ring. The strongest signal at 123 ppm is principally assigned to the un-substituted phenyl carbon atom. Meanwhile, both relatively weaker signals at 106 and 137 ppm could be caused by the substituted phenyl carbon atom [47]. This evidence indicate that JJU-1 possesses the objective skeleton. On the other hand, powder X-ray diffraction (PXRD) was used to study the crystallinity of as-synthesized materials. No obvious diffraction peak is found in Figure 1c, suggesting the amorphous nature of JJU-1. As illustrated in Figure 1d, the thermogravimetric analysis (TGA) curve shows that this prepared sample has high thermal stability in air.

**Figure 2:**
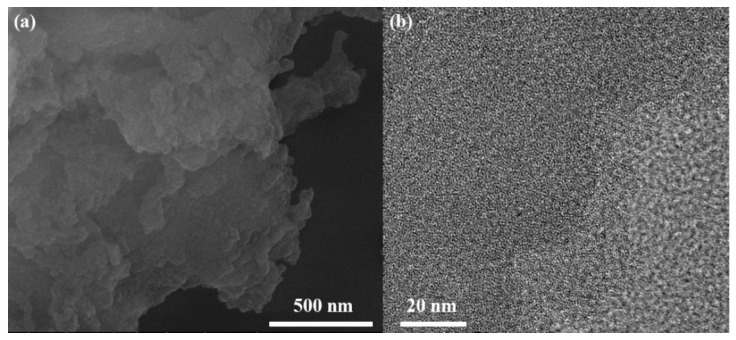
(**a**) SEM and (**b**) TEM images of the as-synthesized JJU-1.

Furthermore, scanning electron microscopy (SEM) and transmission electron microscopy (TEM) technologies have been widely used to study the morphology and microstructure. Herein, SEM and TEM images were recorded for inspecting the morphology and structure of as-synthesized JJU-1. As seen in (Figure 2a), the SEM image shows that the prepared solid samples are cross-linked irregular nanoparticles, which is consistent with the PXRD pattern. In addition, the TEM still exhibits that JJU-1 has an out-of-order and worm-like porous structure (Figure 2b). According to these results, the as-synthesized JJU-1 is successfully synthesized as amorphous powder materials.

### 2.2. Gas Sorption Properties

**Figure 3:**
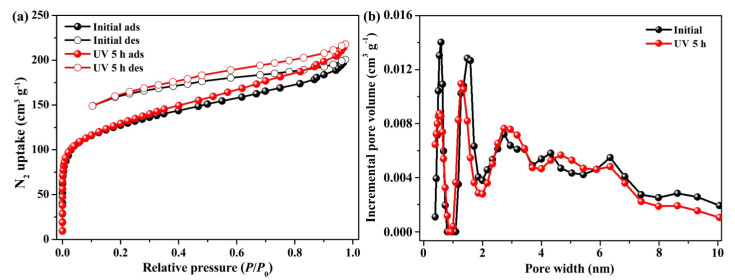
(**a**) N_2_ adsorption and desorption isotherms and (**b**) pore size distributions of initial JJU-1 (black) and after UV irradiation for 5 h (red).

N_2_ sorption isotherms of activated samples after heating at 150 °C for 10 h under vacuum were measured at 77 K to investigate the porous structure of JJU-1. As shown in (Figure 3a), the adsorption isotherms are the combination phenomena of type-I and type-IV curves [48], which is widely found in some reported POF materials [49,50,51]. The N_2_ adsorption amount can rapidly reach up to 115.9 cm^3^ g^–1^ at 0.1 atm, and the maximum N_2_ uptake is as high as 200.6 cm^3^ g^–1^ at 1 atm. The N_2_ sorption performance mainly happens in the micropores, as the sharp adsorption amounts at the low pressure region. Besides, the pore expansion and larger pores cause the distinct hysteresis between the adsorption and desorption plots. Furthermore, the increasing sorption uptake with the boosting pressure proves the existence of mesoporous structure. The surface area of activated JJU-1 is calculated by Langmuir and Brunauer–Emmet–Teller (BET) models. Meanwhile, the pore size distribution of JJU-1 is calculated by the non-local density functional theory (NLDFT). All results are summarized and listed in (Table 1). JJU-1 displays different N_2_ sorption behaviors at 77 K after UV light irradiation for 5 h, with a slightly increasing N_2_ sorption amount (Figure 3a). The BET surface area plots are shown in Figure S2 and Table S1. Although the specific surface area is similar with the initial sample, it is worth mentioning that the micropore size distribution of JJU-1 decreases slightly after UV light irradiation (Figure 3b). In addition, the pore volume of JJU-1 also slightly changes after UV light irradiation. These results prove that the introduction of azobenzene into the porous skeleton is an efficient approach for regulating the pore property of POFs.

**Figure 4:**
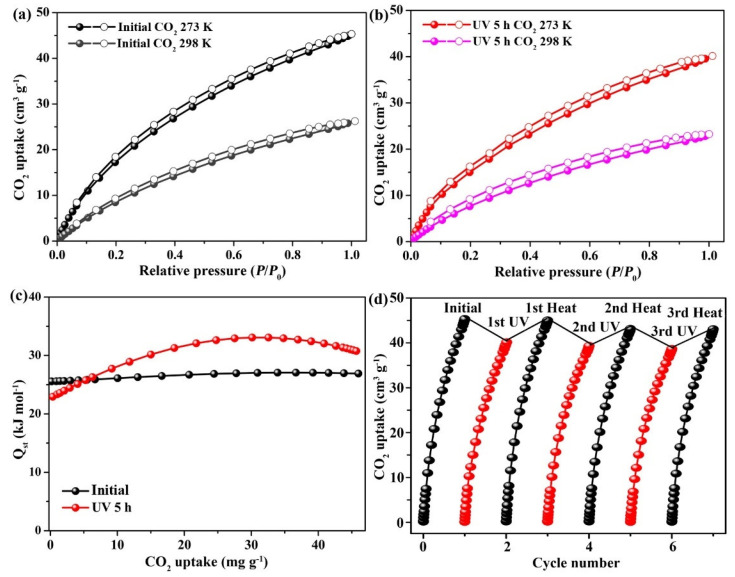
CO_2_ adsorption isotherms of (**a**) initial JJU-1 and (**b**) JJU-1 after UV irradiation for 5 h; (**c**) the corresponding Q_st_ plots; (**d**) the reversibility of JJU-1 after UV and heat treatment for three cycles.

According to previous reports, the *trans*/*cis* isomerization of azobenzene is able to generate apparent geometrical and dipole changes [52,53,54,55]. CO_2_ sorption tests were carried out to obtain the uptake capacity, which can be used to investigate the dynamic photoswitching for controllable CO_2_ adsorption of JJU-1. As seen in (Figure 4a), the initial JJU-1 can obviously adsorb a large amount of CO_2_ at 273 and 298 K at 1 atm, with maximum uptakes of 45.3 and 26.3 cm^3^ g^–1^. Nevertheless, JJU-1 after UV irradiation for 5 h shows significantly different CO_2_ adsorption behaviors (Figure 4b). Furthermore, the CO_2_ adsorption enthalpy (Q_st_) can be calculated based on CO_2_ sorption isotherms at 273 K and 298 K (Figure 4c and Figure S3). The Q_st_ value of the initial sample toward CO_2_ is calculated as 27.1 kJ mol^–1^. The Q_st_ value of JJU-1 after UV irradiation for 5 h is obviously different from that of initial samples; meanwhile, the corresponding Q_st_ value increases to 33.1 kJ mol^–1^. The configuration change of azobenzene from *trans* to *cis* results in an increased affinity for CO_2_; however, the reduction of micropore volume is the major reason for the decreasing CO_2_ adsorption after UV irradiation. More importantly, the CO_2_ sorption isotherms of the material after UV irradiation and thermal regeneration were repeatedly measured for three cycles. Figure 4d and Figure S4 show the detailed CO_2_ adsorption isotherms at 273 K under 1 atm. No obvious variation of the CO_2_ adsorption behavior was observed during the *trans*/*cis* isomerization of the azo-functional skeleton after recycling three times via alternating external stimuli (Table 2). Comparison with the previous reported materials, this prepared JJU-1 exhibits outstanding CO_2_ sorption performance under controlled external stimuli (Table S2). Additionally, the gas sorption behaviors may be mainly caused by the reversible *trans*/*cis* transformation of the azo functional group under UV irradiation and thermal regeneration (Figure S5). Three UV irradiation/thermal relaxation cycles confirm the reversible behavior during the CO_2_ storage/release process. As a result, the introduction of the azo group in POFs is beneficial to the synthesis of photoresponsive POFs toward CO_2_.

## 3. Materials and Methods

### 3.1. Materials and General Methods

All available chemicals were purchased commercially and used as received without further purification. Notably, CHCl_3_ was dried over CaH_2_ before using in the synthetic process. UV-Vis spectra were recorded on Mapada V-1200 (Shanghai Meipuda instrument Co., LTD, Shanghai, China). The UV exposure experiments for *trans* to *cis* isomerization were performed by a ZF-1A UV analyzer (JIAPENG, Shanghai, China). The FT-IR spectra were recorded on an IFS 66V/S Fourier transform infrared spectrometer (Bruker Corporation, Rheinstetten, Germany). The ^13^C CP-MAS spectrum was recorded in a Bruker AV-400-WB (Bruker Corporation, Billerica, MA, USA). PXRD was performed on a Rigaku D/MAX 2550 diffractometer (Rigaku, Tokyo, Japan) by using Cu-Kα radiation at 40 kV and 200 mA over a 2θ range of 4–40^o^. SEM images were measured on a MIRA-3 LMU scanning electron microscope (Tescan, Brno, The Czech Republic). TEM images were collected on a Tecnai G^2^ F20 S-TWIN (FEI, Hillsboro, WA, USA). TGA was implemented on a PerkinElmer STA6000 thermal analyzer (PERKINELMER, Waltham MA, USA) in air at a heating rate of 10 °C min^–1^. Gas sorption measurements were measured on a Micromeritics ASAP 2020 M surface at 77 K (Micromeritics Instrument Corporation, Norcross, GA, USA). For the *cis* to *trans* isomerization, the degassing port on a Micromeritics ASAP 2020 gas sorption analyser was used by setting the program to 150 °C for 10 h.

### 3.2. Synthesis of JJU-1

A mixture of 1,3-*bis*(N-carbazolyl)benzene (204 mg, 0.5 mmol), azobenzene (456 mg, 2.5 mmol), and FeCl_3_ (487 mg, 3 mmol) was added in a round-bottomed flask. After pumping into a vacuum, the reaction system was trice inflated with inert N_2_. Then, dried CHCl_3_ (20 mL) was added through a syringe. The mixture was heated to 80 °C under N_2_ atmosphere for 24 h. After cooling to room temperature, the crude product was obtained by filtration and washed with water, chloroform, methanol and acetone to remove unreacted monomers or catalyst residues. Further purification of as-synthesized JJU-1 was carried out by the Soxhlet extraction with methanol for 48 h. The final product was dried in a vacuum for 6 h at 60 °C to give the target samples.

## 4. Conclusions

In this work, an azo-containing POF has been successfully synthesized via FeCl_3_-promoted oxidative coupling polymerization. The structure and porosity of the prepared material are well characterized and discussed. The *trans*/*cis* isomerization of azo group in the porous skeleton is achieved by UV irradiation and thermal regeneration. The CO_2_ uptake corresponds to a 11.5% decrease after UV irradiation, and almost recovers to the initial value after thermal regeneration. Furthermore, the CO_2_ adsorption isotherms of the POF using UV irradiation and thermal regeneration are repeatedly measured for three cycles. This work proves that responsive azo-functionalized POFs with controllable adsorption of CO_2_ have great potential applications in CCS, and are worthy of further study.

## Figures and Tables

**Table 1:** Summary of porosity, pore volume and CO_2_ adsorption properties for JJU-1.

Samples	SA_BET_^a^ [m^2^ g^–1^]	SA_Langmuir_^b^ [m^2^ g^–1^]	V_Total_^c^ [cm^3^ g^–1^]	V_Micro_^d^ [cm^3^ g^–1^]	CO_2_ Uptake [cm^3^ g^–1^]		Q_st_ CO_2_ (kJ mol^–1^)
					**273 K**	**298 K**	
Initial	467	530	0.31	0.15	45.3	26.3	27.1
UV 5 h	469	532	0.34	0.17	40.1	23.2	33.1

^a^ Surface area (m^2^ g^−1^) calculated from the N_2_ adsorption based on the BET model; ^b^ Surface area (m^2^ g^−1^) calculated from the N_2_ adsorption isotherms based on the Langmuir model; ^c^ The total pore volume (cm^3^ g^−1^) calculated at *P*/*P*_0_ = 0.97; ^d^ The micropore volume (cm^3^ g^−1^) calculated by the t-plot method.

**Table 2:** Summary of CO_2_ adsorption properties of JJU-1.

JJU-1	CO_2_ Uptake (273 K, 1 bar), [cm^3^ g^−1^]
Initial	45.3
1st UV	40.1
1st Heat	45.0
2nd UV	39.3
2nd Heat	43.1
3rd UV	38.7
3rd Heat	43.1

## Data Availability

The data presented in this study are available in the Supplementary Materials.

## Supplementary Materials

The following are available online, Figure S1: Changes in the adsorption spectra of azobenzene in CH_2_Cl_2_ over the time during the irradiation with 365 nm light; Figure S2: BET surface area plots; Figure S3: The plots of pressures in function of gas uptakes and the parameters (virial-type expression) for the calculation of heats of adsorption of CO_2_ for (a) initial JJU-1 and (b) JJU-1-1st UV; Figure S4: The reversible CO_2_ adsorption isotherms at 273 K by UV irradiation and thermal treatment of JJU-1; Figure S5: The schematic diagram of the structure changes during the reversible *trans/cis* transformation of azo functional group under UV irradiation and thermal regeneration; Table S1: The BET surface area report of JJU-1; Table S2: BET surface area reports and CO_2_ uptakes of some similar POFs.

## Sample Availability

Samples of the compounds are Not available from the authors.
