# Supplementary material for: Rational Construction of a Responsive Azo-Functionalized Porous Organic Framework for CO2 Sorption
Source: Molecules. 2021 Aug 18;26(16):4993. doi: 10.3390/molecules26164993 (PMC8398054; doi:10.3390/molecules26164993)
Supplement: Supplementary file 1 [file molecules-26-04993-s001.zip › molecules-1337751-supplementary.pdf]

Supporting Information

Rational Construction of a Responsive Azo-Functionalized Porous Organic Framework for CO_2 Sorption

Rongrong Yuan [1], Hao Sun [1] and Hongming He [2,*]

[1] Department of Materials Science and Engineering, Jilin Jianzhu University, Changchun 130118, P. R. China

[2] College of Chemistry, Tianjin Key Laboratory of Structure and Performance for Functional Molecules, Tianjin Normal University, Tianjin 300387, P. R. China

Figure S1. Changes in the adsorption spectra of azobenzene in CH_2Cl_2 over the time during the irradiation with 365 nm light

Figure S2. BET surface area plots

Table S1. The BET surface area report of JJU-1

Figure S3. The plots of pressures in function of gas uptakes and the parameters (Virial-type expression) for the calculation of heats of adsorption of CO_2 for (a) initial JJU-1 and (b) JJU-1-1st UV

Figure S4. The reversible CO_2 adsorption isotherms at 273 K by UV irradiation and thermal treatment of JJU-1

Figure S5. The schematic diagram of the structure changes during the reversible *trans/cis* transformation of azo functional group under UV irradiation and thermal regeneration

Table S2. BET surface area reports and CO_2 uptakes of some similar POFs

References

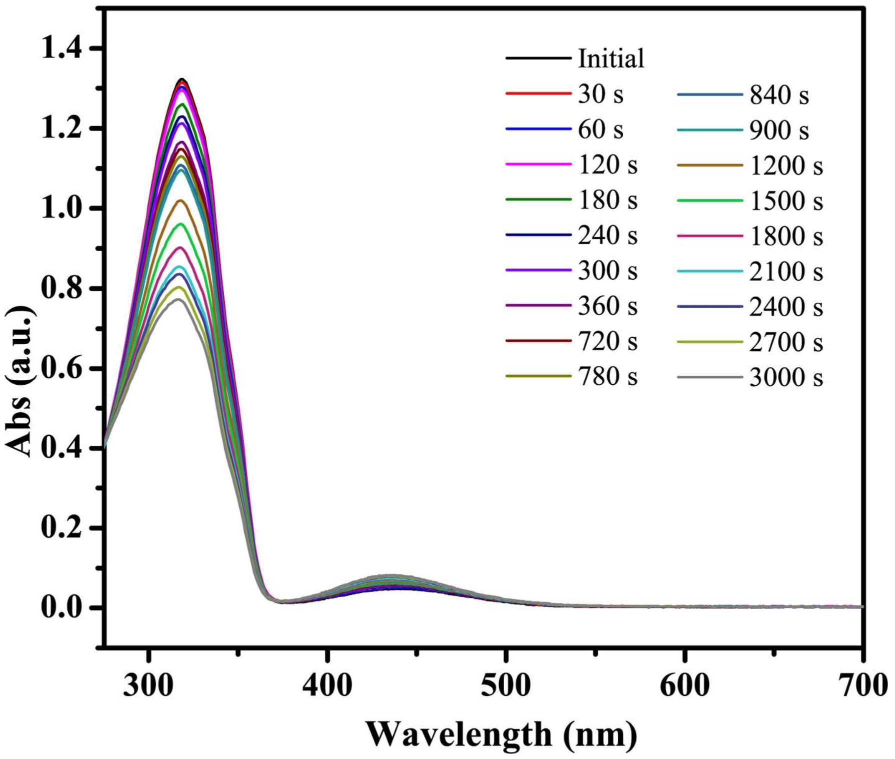

Figure S1. Changes in the adsorption spectra of azobenzene in CH_2Cl_2 over the time during the irradiation with 365 nm light.

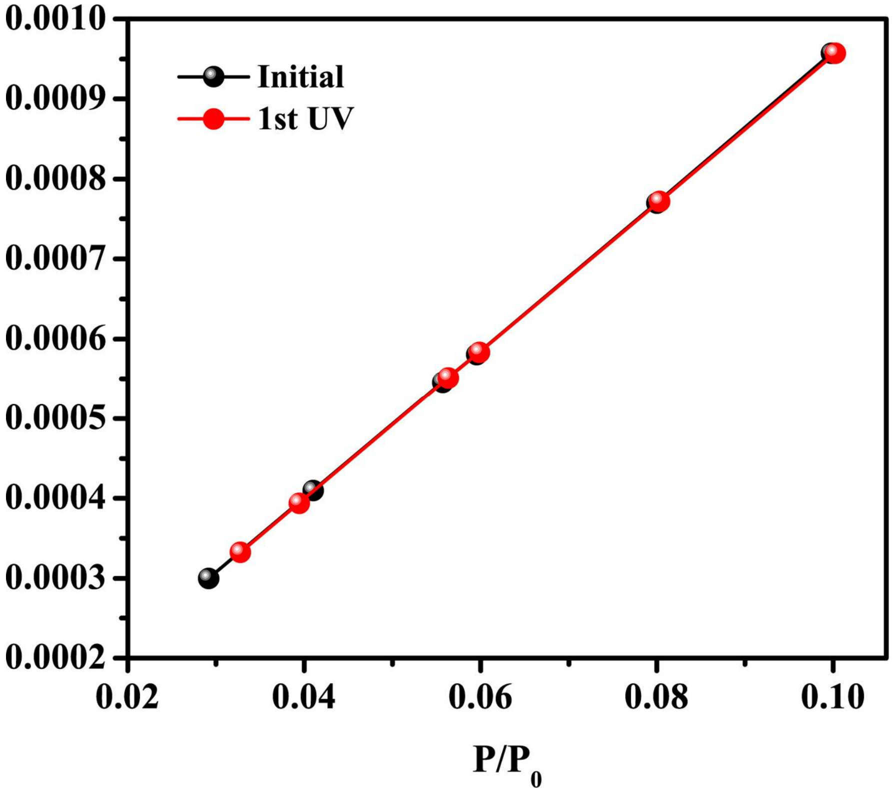

Figure S2. BET surface area plots.

Table S1. The BET surface area report of JJU-1.

**JJU-1**	**BET [m² g⁻¹]**	**Slope [g cm⁻³]**	**Y-intercept [g cm⁻³]**	**C**	**Qm [cm³ g⁻¹]**	**Correlation coefficient**
**Initial**	467.09	0.009290	0.000028	327.398390	107.3127	0.9999919
**1st UV**	468.68	0.009258	0.000029	318.840491	107.6778	0.9999978

Calculation of isosteric heat of adsorption

The CO_2 isosteric heat of adsorption of JJU-1 was calculated as a function of the adsorption isotherms at 273 K and 298 K. The date were modelled with a virial-type expression composed of parameters a_i and b_i (eq 1), and the heat of adsorption was calculated from the fitting parameters using eq 2, where P is the pressure, N is the amount adsorbed, T is the temperature, R is the universal gas constant, and m and n determine the number of terms required to adequately describe the isotherm. a_i and b_i are virial coefficients.

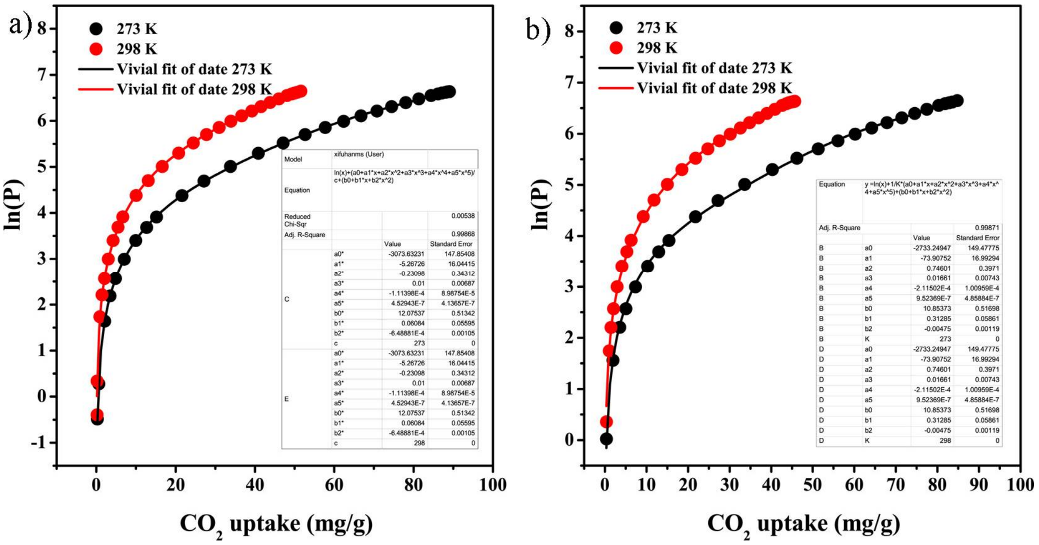

Figure S3. The plots of pressures in function of gas uptakes and the parameters (Virial-type expression) for the calculation of heats of adsorption of CO_2 for (a) initial JJU-1 and (b) JJU-1-1st UV.

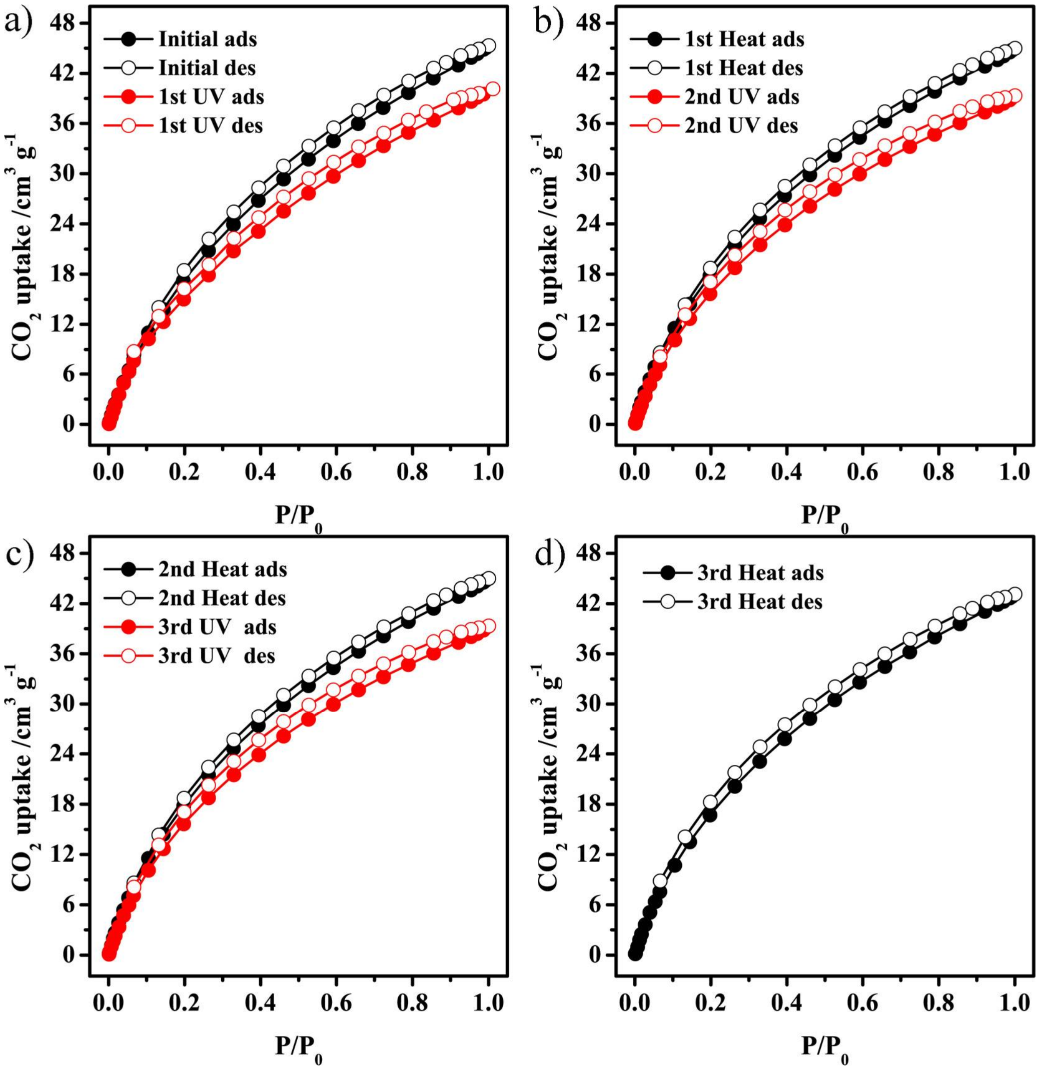

Figure S4. The reversible CO_2 adsorption isotherms at 273 K by UV irradiation and thermal treatment of JJU-1.

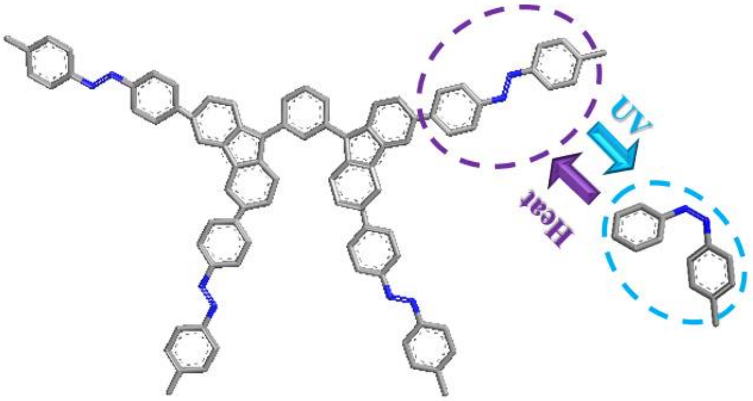

Figure S5. The schematic diagram of the structure changes during the reversible *trans*/*cis* transformation of azo functional group under UV irradiation and thermal regeneration.

Table S2. BET surface area reports and CO_2 uptakes of some similar POFs.

Materials	BET [m^2/g]	V_{total} [cm^3/g]	CO_2 uptake [cm^3/g]	Q_{st} [kJ/mol]	Reference
**Azo-MOP-1**	456	0.48	49.6	--	1
**PAF-36**	325	0.25	28.6	27.0	2
**PAF-36-UV**	385	0.27	31.2	28.4	2
**PAF-37**	443	0.27	26.3	36.8	2
**PAF-37-UV**	456	0.28	29.6	40.7	2
**TAP-1**	474	0.74	48.4	35.6	3
**UCBZ-1**	980	1.46	39.0	--	4
**UCBZ-1-UV**	--	--	50.0	--	4
**azo-COP-1**	608	0.39	54.7	29.3	5
**azo-COP-2**	703	0.42	56.2	24.8	5
**Azo-POF-1**	712	--	66.7	27.5	6
**Azo-POF-2**	439	--	43.0	26.6	6
**ALP-4**	862	0.50	41.2	28.2	7
**POF-Initial**	571	0.49	46.2	26.7	8
**POF-1st UV**	549	0.48	41.6	29.7	8
**JJU-1-Initial**	467	0.31	45.3	27.1	This work
**JJU-1st UV**	469	0.34	40.1	33.1	This work

References

[1] Yang, Z.; Zhang, H.; Yu, B.; Zhao, Y.; Ma, Z.; Ji, G.; Han, B.; Liu, Z. Azo-Functionalized Microporous Organic Polymers: Synthesis and Applications in CO_2 Capture and Conversion. *Chem. Commun*. **2015**, *51*, 11576–11579.

[2] Yuan, R.; Ren, H.; He, H.; Jiang, L.; Zhu, G. Targeted Synthesis of Porous Aromatic Frameworks with Stimuli-Responsive Adsorption Properties. *Sci. China Mater.* **2015**, *58*, 38–43.

[3] Bera, R.; Ansari, M.; Alam, A.; Das, N. Triptycene, Phenolic-OH, and Azo-Functionalized Porous Organic Polymers: Efficient and Selective CO_2 Capture. *ACS Appl. Polym. Mater*. **2019**, *1*, 959−968.

[4] Zhu, Y.; Zhang, W. Reversible Tuning of Pore Size and CO_2 Adsorption in Azobenzene Functionalized Porous Organic Polymers. *Chem. Sci*. **2014**, *5*, 4957–4961.

[5] Patel, H.A.; Je, S.H.; Park, J.; Chen, D.P.; Jung, Y.; Yavuz, C.T.; Coskun, A. Unprecedented High-Temperature CO_2 Selectivity in N_2-Phobic Nanoporous Covalent Organic Polymers. *Nat. Commun*. **2013**, *4*, 1357.

[6] Lu, J.; Zhang, J. Facile Synthesis of Azo-Linked Porous Organic Frameworks via Reductive Homocoupling for Selective CO_2 Capture. *J. Mater. Chem. A* **2014**, *2*, 13831–13834.

[7] Arab, P.; Rabbani, M.G.; Sekizkardes, A.K.; Islamoglu, T.; El-Kaderi, H.M. Copper(I)-Catalyzed Synthesis of Nanoporous Azo-Linked Polymers: Impact of Textural Properties on Gas Storage and Selective Carbon Dioxide Capture. *Chem. Mater.* **2014**, *26*, 1385–1392.

[8] Yuan, R.; Sun, H.; Yan, Z.; He, H. Rational Design and Synthesis of a Task-Specific Porous Organic Framework Featured Azobenzene as a Photoresponsive Low-Energy CO_2 Adsorbent. *J. Solid State Chem.* **2021**, *297*, 122049.
